# Regulation of AMH, AMHR-II, and BMPs (2,6) Genes of Bovine Granulosa Cells Treated with Exogenous FSH and Their Association with Protein Hormones

**DOI:** 10.3390/genes10121038

**Published:** 2019-12-12

**Authors:** Saqib Umer, Abdul Sammad, Huiying Zou, Adnan Khan, Bahlibi Weldegebriall Sahlu, Haisheng Hao, Xueming Zhao, Yachun Wang, Shanjiang Zhao, Huabin Zhu

**Affiliations:** 1 Embryo Biotechnology and Reproduction Laboratory, Institute of Animal Sciences, Chinese Academy of Agricultural Sciences, Beijing 100193, China; saqibumar33@hotmail.com (S.U.); zouhuiying@caas.cn (H.Z.); blenbah@gmail.com (B.W.S.); haohaisheng@caas.cn (H.H.); zhaoxueming@caas.cn (X.Z.); zhaoshanjiang@caas.cn (S.Z.); 2 Key Laboratory of Animal Genetics, Breeding and Reproduction, CAST, China Agricultural University, Beijing 100193, China; drabdulsammad1742@yahoo.com (A.S.); dr.adnan93@cau.edu.cn (A.K.); wangyachun@cau.edu.cn (Y.W.)

**Keywords:** AMH, BMPs, bovine granulosa cells, FSH treatment, hormone

## Abstract

Anti-Mullerian hormone (AMH) is an important reproductive marker of ovarian reserve produced by granulosa cells (GCs) of pre-antral and early-antral ovarian follicles in several species, including cattle. This hormone plays a vital role during the recruitment of primordial follicles and follicle stimulating hormone (FSH)-dependent follicular growth. However, the regulatory mechanism of AMH expression in follicles is still unclear. In this study, we compared the expression of *AMH, AMHR-II, BMP2, BMP6, FSHR,* and *LHCGR* genes during follicular development. In-vitro expression study was performed with and without FSH for *AMH, AMHR-II, BMP2,* and *BMP6* genes in bovine GCs which were isolated from 3–8 mm follicles. Association among the mRNA expression and hormone level was estimated. GCs were collected from small (3–8 mm), medium (9–12 mm) and large size (13 to 24 mm) follicles before, during onset, and after deviation, respectively. Further, mRNA expression, hormones (AMH, FSH, and LH), apoptosis of GCs, and cell viability were detected by qRT-PCR, ELISA, flow cytometry, and spectrophotometry. *AMH, AMHR-II, BMP2,* and *FSHR* genes were highly expressed in small and medium follicles as compared to large ones. In addition, the highest level of AMH protein (84.14 ± 5.41 ng/mL) was found in medium-size follicles. Lower doses of FSH increased the viability of bovine GCs while higher doses repressed them. In-vitro cultured GCs treated with FSH significantly increased the *AMH, AMHR-II,* and *BMP2* expression levels at lower doses, while expression levels decreased at higher doses. We found an optimum level of FSH (25 ng/mL) which can significantly enhance *AMH* and *BMP2* abundance (*p* < 0.05). In summary, *AMH, AMHR-II,* and *BMP2* genes showed a higher expression in follicles developed in the presence of FSH. However, lower doses of FSH demonstrated a stimulatory effect on *AMH* and *BMP2* expression, while expression started to decline at the maximum dose. In this study, we have provided a better understanding of the mechanisms regulating *AMH, AMHR II,* and *BMP2* signaling in GCs during folliculogenesis, which would improve the outcomes of conventional assisted reproductive technologies (ARTs), such as superovulation and oestrus synchronization in bovines.

## 1. Introduction

In bovines, MOET (multiple ovulation and embryo transfer) has been under practiced for many years. MOET has the potential to increase the genetic improvement rate and production of beef and dairy cattle breeds. Recent advances in genomic selection have brought dramatic changes in the genetic screening programs, but in cattle, lower reproductive efficiency is a significant constraint in the rate of genetic improvement [1,2]. Among cows, the ovarian response of FSH (follicle stimulating hormone) superstimulation is quite variable, and similar problems are persistent in humans. This is a major limiting factor for successful embryo production [3,4]. In cattle, reproductive ultrasonography is helpful in the prediction of superovulation success, such as the presence of preovulatory and periovulatory follicles [5,6], opening the window for new protocols of in vivo embryo production [7]. The process by which a single follicle becomes dominant and a cohort undergoes atresia in bovines is regulated by many intrafollicular factors, belonging to the transforming growth factor-β (TGF-β) superfamily. However, this family consists of BMPs which play a significant role in the growth and differentiation of granulosa cells (GCs), and resultantly produce a dominant follicle [8]. Anti-Mullerian hormone (AMH), a member of the TGF-β family, composed of 551 amino acids, is released by GCs of preantral and antral follicles [9]. AMH could serve as a potential reproductive biomarker for the prediction of the ovarian reserve pool of follicles in bovines [10,11]. AMH plays a vital role by inhibiting primordial follicle recruitment and dominant follicle selection during follicular waves via paracrine factors, and it reduces the follicular sensitivity to FSH [12]. Hence, AMH can mediate ovarian folliculogenesis and follicular sensitivity [13,14]. Moreover, the blood circulating concentration of AMH has been suggested to be used to predict the success of assisted reproductive technologies (ARTs; including ovarian superstimulation, in vitro embryo production, embryo transfer and ovarian disorders) [15,16,17]. AMH also requires ligand-based receptor type-II (AMHR-II) to play its role without sharing the receptor to other TGF-β family members. It binds with AMHR-II and simultaneously dimerizes with type-I, resultantly activating downstream transcription factors (*SMAD1*, *SMAD5*, *and SMAD8*) [18,19,20,21]. Recently, various studies have revealed that BMPs (bone morphogenetic proteins) may regulate AMH via the SMAD pathway [22,23]. The involvement of the BMP system has been well known for the regulation of ovarian functioning in many animals, including rats and cattle [24,25]. BMPs, primarily BMP2, BMP4, BMP6, and BMP7, play a key role in female fertility by regulating the proliferation of GCs and steroidogenesis [26]. BMPs are target-specific for every type of cell, especially BMP2 and BMP6 which are expressed mainly in GCs, whereas FSH based progesterone synthesis is inhibited by BMP ligands [25,27,28,29,30].

In the current study, FSH is hypothesized to have an inverse impact on AMH, AMHRII, and BMPs (2 and 6), which can mediate inhibitory signaling to suppress premature and dominant follicle selection. In this study, we compared the expression of *AMH, AMHR-II, BMP2, BMP6, FSHR,* and *LHCGR* genes during follicular development. In-vitro expression study was performed with and without FSH for *AMH, AMHR-II, BMP2,* and *BMP6* genes in bovine GCs which were isolated from 3–8 mm follicles. The capability of FSH to repress premature bovine GCs livability and apoptosis was also assessed. The association between mRNA expression and hormone level was estimated. We predicted that a better understanding of the mechanisms regulating AMH and/or AMHR II (related transcript) signaling in GCs during follicle development would ultimately improve the outcomes of conventional assisted reproductive technologies (ARTs) such as superovulation treatments and oestrus synchronization protocols in bovines.

## 2. Material and Methods

### 2.1. Animals and Tissue Collection

Ovaries were collected from adult, cyclic, and non-pregnant cows, irrespective of the stage of oestrus cycle, at a local abattoir. After exsanguination, ovaries were placed in normal saline (NS; 1% Antibiotic; temp 37 °C) and shifted to the laboratory within one hour. All the ovaries were visually examined for any structural abnormality. They were washed with NS (3–5 times) and phosphate buffer solution (PBS) 3 times prior to collecting GCs.

### 2.2. Granulosa Cells and Follicular Fluid Collection

Follicles were categorized according to size and follicle development, i.e., small (3–8 mm; pre deviation or undifferentiated), medium (9–12 mm; onset of deviation) and large (13–24 mm; post deviation). GCs were isolated by a 23 G needle and pooled together with follicular fluid (FF) and sieved through 40 um filter (Falcon Corning, New York, USA) and then centrifuged (1500 rpm for 5 min). Later on, the supernatant follicular fluid was separated, and GCs were washed with PBS (2 times; 1500 rmp for 5 min). Afterward, GCs and FF were stored at −80 °C for further analysis.

### 2.3. Culturing of Granulosa Cells

For culturing, fresh mural granulosa cells were isolated from small follicles (3–8 mm) and seeded at 2 × 10^6^ cells/well in 6-well polystyrene culture plate (Costar^®^, Corning Incorporated-life Science, Shanghai, China). GCs were washed 2 times with PBS (without Ca^2+^ and Mg^2+^; Biological Industries, USA) and centrifuged at 1500 rpm for 5 min. DMEM/F12 medium (Dulbecco’s Modified Eagle Medium Nutrient mixture F-12 Hem; Gibco^®^, New York, USA) was used for all in vitro experiments, containing 10% FBS (Fetal Bovine Serum; Gibco^®^, New York, USA) and 1% Antibiotic (100 U/mL Penicillin and 100 ug/mL Streptomycin) at 37 °C with 5% CO_2_ (Carbon dioxide) and 95% Air maintained, with or without FSH (1, 5, 10, 25 and 50 ng/mL; Sigma-Aldrich, St. Louis USA ). Afterward, cells were cultured for 72 h. For the first 24 h, all the cells were cultured in the same medium (DMEM/F12) to check the purity and viability as described by Lerner et al. [31]. Subsequently, the medium was changed and cells cultured in the absence or presence of FSH (1, 5, 10, 25 and 50 ng/mL) for 48 h in total volume 2 mL/well to evaluate the expression effect on *AMH*, *AMHR II*, *BMP2*, *BMP6*, and *CYP19A1*. Each test was repeated in triplicate related to cultured GCs. At the end of culturing, the medium was stored at −80 °C for the AMH assay. The cells were detached with trypsin (0.25%) and shifted to sterile Eppendorf tubes for washing with PBS by centrifugation. Later, the cells were stored at −80 °C till further use. 

### 2.4. Cell Proliferation Assay

A cell proliferation assay was performed with a Cell Counting Kit-8 (CCK-8; BBI life Science, Sangon Biotech, Shanghai China) as per manufacturer protocol. Briefly, 2 × 10^6^ GCs were added in each well. The culture medium, without GCs, was used as a negative control. The culture medium with GCs was used as control. The treatment medium with GCs was used as treatment group, and five wells were used for every single sample. At the end of culture, 10 µL CCK-8 reagent was added to each well and incubated at room temperature for 30 min. The absorbance of cultured GCs was measured by infinite M200 PRO (TECAN, Germany) at 450 nm. The data represented five independent cultures with each treatment considered as quintuplicate.

### 2.5. Apoptotic Assay

The qualitative and quantitative apoptosis of GCs was assessed by fluorescence microscope and flow cytometry with an Annexin V-FITC (njjcbio, Nanjing, China), respectively. GCs were harvested by 0.25% trypsin after varying doses of FSH. Later harvesting, GCs were washed twice with PBS and then added 500 ul 1× binding buffer. Subsequently, 5 ul FITC and PI were added. After 20 min incubation at room temperature, slides were prepared and observed under a fluorescence microscope (Axio Imager A2, Zeiss Germany).

For the quantitative apoptotic assay, samples were prepared again as aforementioned and flow cytometry was performed by FACS Calibur (BD Biosciences, CA, USA) and data were analyzed by Flowjo 10 software.

### 2.6. RNA Purification, cDNA Preparation and Real-Time Quantitative PCR (qRT-PCR)

After collection (different size follicles) and Culturing (with or without FSH) of GCs, washed with PBS and used for total RNA extraction. RNeasy Mini Kit (QIAGEN, Germany; Cat log # 74104) used for RNA extraction as per the manufacturer’s protocol. We quantified the total RNA by NanoPhotometer N50 Touch (IMPLEN GMBH, Germany). cDNA (Complementary DeoxyriboNucleic Acid) was prepared by using 2 µg RNA, 1 µL Oligo(dT)_18_ Primer (Thermo scientific, Europe) and water-DEPC (Sangon Biotech, Shanghai, China) to make 15 µL total volume then put in Proflex PCR machine (temperature 75 °C, 5 min, single cycle; applied biosystem^®,^ Singapore). Afterward, 1 µL dNTP Mix 10 mM (invitrogen, USA), 1 µL recombinant RNase Inhibitor (TaKaRa, Shiga, Japan), 1 µL reverse transcriptase M-MLV (RNase H-; TaKaRa, Shiga, Japan), 5 µL 5× M-MLVT Buffer (TaKaRa, Shiga, Japan) and 2 µL water-DEPC were added and then put in Proflex PCR machine (temperature 42 °C, 60 min, single cycle). 

The qRT-PCR reactions were set-up by using a CFX96 Real-Time System (Bio-Rad Laboratories, Inc. CA, USA). A total 15 µL volume was used for performing amplification reaction, containing 2 µL cDNA, 0.35 µL each forward and reverse primers, 7.5 µL SYBR and 4.8 µL nuclease-free water. Cycle amplifications were comprised of an initial denaturation step at 95 °C for 10 min, followed by 39 cycles at 95 °C for 15 sec and 60 °C for 60 sec. The relative expression of nominated genes was calculated by 2-ΔΔCt method [32]. Specific primer sequences were synthesized by Sangon Biotech (Beijing, China). The primer sequences used for qRt-PCR are shown in Table 1.

### 2.7. Hormonal Assay

After collecting GCs, the intrafollicular fluid was stored at −80 °C for the determination of AMH, FSH, and LH. Similarly, the medium after culturing (FSH treatment) was stored for AMH assay. All commercial ELISA kits were purchased from CUSABIO TECHNOLOGY LLC. The concentrations of AMH (Cat no. CSB-YP001666BO1), FSH (Cat no. CSB-E15856B), and LH (Cat no. CSB-E12826B) were determined according to manufacturer protocols. Each treatment was performed in triplicate. The absorbance was measured by infinite M200 PRO (TECAN, Germany) at 450 nm.

### 2.8. Statistical Analysis

The data were subjected to statistical analysis using IBM SPSS (Statistical Package for Social Sciences) software version 26.0 Armonk, NY, USA. Analysis of variance was performed, and means were compared using Tukey’s honestly significant difference (HSD) test at 5% level of significance (α = 0.05). The correlation between different variables was analyzed using Pearson’s correlation co-efficient with regression analysis at a 5% level of significance using RStudio software version 1.2.5001.

## 3. Results

GCs were collected from different sized follicles based upon follicle deviation. Analyses considered the undifferentiated status of GCs of 3–8 mm follicles (small), onset of deviation from nine to 12 mm follicles (medium), and post deviation, or luteinized GCs from 13–24 mm follicles (large). We used two reference genes, *GAPDH* and *ACTB*, while relative expression in the whole experiment were standardized with *GAPDH* (*p* < 0.05). All the data represented in figures are expressed as mean ± S.E. Data were normalized for graphical representation of the small size follicle group and cultured undifferentiated GCs with the control group. To confirm the abundance of GC specific transcriptome, we measured *CYP19A1* expression owing to the fact that the GC-specific gene is involved in the steroidogenic pathway.

### 3.1. Abundance and Regulation of FSHR and LHCGR in Bovine GCs

**Figure 1:**
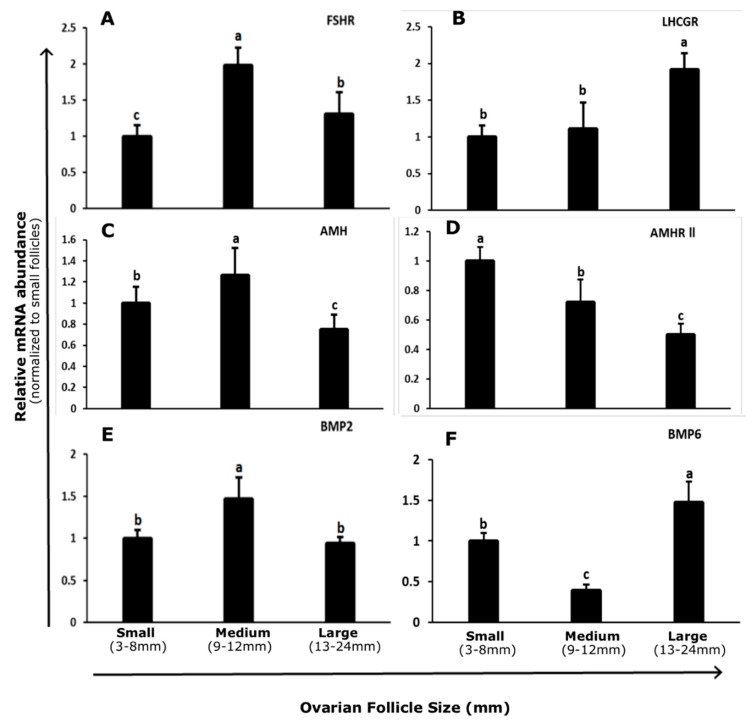
Transcript abundance of *FSHR* (**A**) *LHCGR* (**B**) *AMH* (**C**) *AMHR-II* (**D**) *BMP2* (**E**) and *BMP6* (**F**) in granulosa cells of different size follicles. Data were standardized to the *GAPDH* reference gene and expressed as fold difference ± standard error of mean compared with 3 to 8 mm follicles. Means without common letters are significantly different (*p* < 0.05; *n* = 5 per follicle size group). *FSHR* = Follicle Stimulating Hormone Receptor. *LHCGR* = Luteinizing Hormone/Choriogonadotropin Receptor. *AMH* = Anti-Mullerian Hormone. *AMHR-II* = Anti-Mullerian Hormone Receptor type-II. *BMP2* = Bone Morphogenetic Protein 2. *BMP6* = Bone Morphogenetic Protein 6.

The abundance of *FSHR* mRNA in bovine GCs was highest in 9–12 mm follicles and lowest in undifferentiated GCs isolated from 3–8 mm follicles (*p* < 0.05; Figure 1A). *LHCGR* mRNA expression in bovine GCs increased with follicle size. The minimum level of transcript abundance was found between 3 to 8 mm follicles, and maximum abundance was between 13 to 24 mm follicles (*p* < 0.05; Figure 1B).

**Figure 2:**
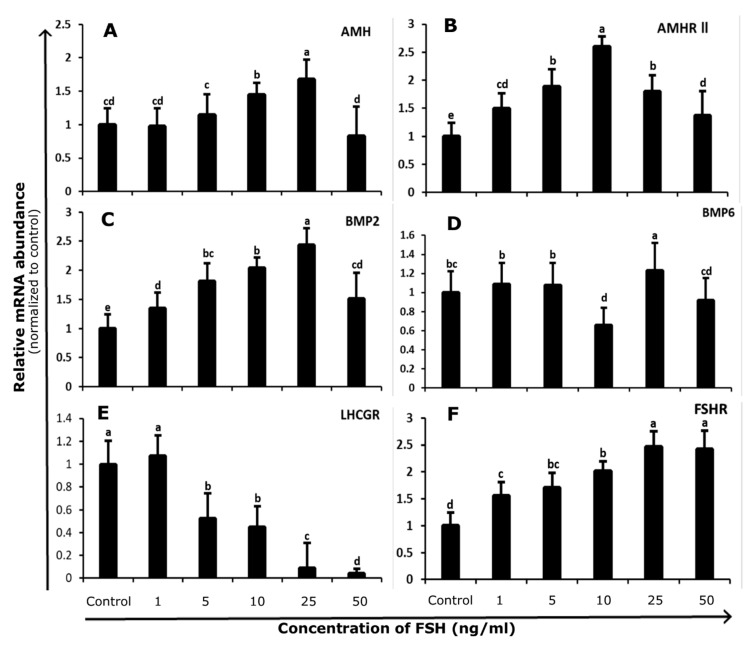
Undifferentiated bovine granulosa cells were isolated from 3 to 8 mm follicles and cultured with or without FSH (0, 1, 5, 10, 25, and 50 ng/mL). The transcript abundance of *AMH* (**A**) *AMHR II* (**B**) *BMP2* (**C**) *BMP6* (**D**) *LHCGR* (**E**)**,** and *FSHR* (**F**) genes in cultured granulosa cells. Data were standardized according to the *GAPDH* reference gene and expressed as fold difference ± standard error of mean compared with untreated control. Means without common letters are significantly different (*p* < 0.05; *n* = 5 independent cultures). *FSHR* = Follicle Stimulating Hormone Receptor. *LHCGR* = Luteinizing Hormone/Choriogonadotropin Receptor. *AMH* = Anti-Mullerian Hormone. *AMHR-I I* = Anti-Mullerian Hormone Receptor type-II. *BMP2* = Bone Morphogenetic Protein 2. *BMP6* = Bone Morphogenetic Protein 6.

Compared to control, FSH stimulation increased *FSHR* (*p* < 0.05; Figure 2E) and decreased *LHCGR* (*p* < 0.05; Figure 2F) transcript levels. The undifferentiated status of 3–8 mm follicle GCs prior and subsequent to culture was confirmed by finding the elevated *FSHR* level and declined *LHCGR* level (near to undetectable).

### 3.2. Abundance and Regulation of AMH and AMHR-II in Bovine GCs

The abundance of *AMH* transcript in bovine GCs was greatest in 9 to 12 mm follicles and decreased in 13 to 24 mm follicles (*p* < 0.05; Figure 1C). However, transcript *AMHR-II* abundance declined with increasing follicle size and a high level of abundance was found in 3–8 mm follicles. The lowest level of abundance was found in 13–24 mm follicles (*p* < 0.05; Figure 1D).

Bovine GCs from 3–8 mm follicles were cultured for 48 h with increasing concentrations of FSH (1, 5, 10, 25, and 50 ng/mL) to evaluate the effects of FSH on GC function. No significant differences in *AMH* mRNA levels were found at 1 and 5 ng/mL FSH doses compared to control. *AMH* mRNA abundance increased upon treatment with 10 and 25ng/mL, while mRNA abundance declined at maximum dose (50ng/mL) and was comparable to the control group (*p* < 0.05; Figure 2A). The transcript abundance of *AMHR-II* was significantly increased till 10 ng/mL FSH (*p* < 0.05; Figure 2B). Meanwhile, at higher concentrations (25 and 50 ng/mL) of FSH, the abundance of *AMHR-II* started to decline. 

### 3.3. Abundance and Regulation of BMP2 and BMP6 in Bovine GCs

The transcript abundance of *BMP2* was significantly increased in 9–12 mm follicles as compared to small (3–8 mm) and large (13–24 mm) follicles. No difference was found between small and large group follicles (*p* < 0.05; Figure 1E), whereas *BMP6* transcript abundance was highest in 13–24 mm and lowest in 9–12 mm follicles (*p* < 0.05; Figure 1F).

In cultures of undifferentiated GCs isolated from 3–8 mm follicles, *BMP2* abundance showed gradual increasing trend up to 25 ng/mL dose. Moreover, *BMP2* abundance decreased at maximum dose (50 ng/mL; *p* < 0.05; Figure 2C). The high level of *BMP6* abundance was found at 25 ng/mL dose and the lowest transcript level at 10 ng/mL treatment (*p* < 0.05; Figure 2D).

### 3.4. Effect of FSH on Survival and Apoptosis of GCs

**Figure 3:**
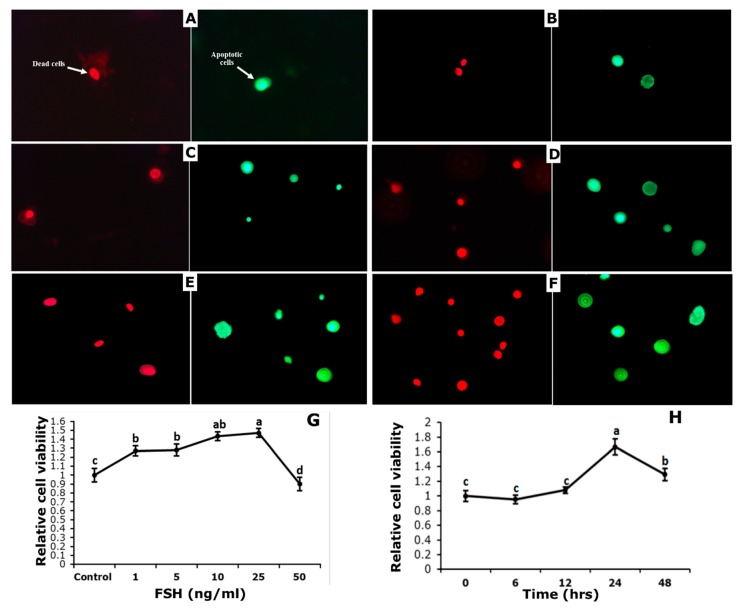
Effect of Follicle Stimulating Hormone (FSH) on apoptosis, death, and viability of cultured granulosa cells. Fluorescence microscope pictures 40X) of dead (red) and apoptotic (green) cells after staining with FITC/PI dye. (**A**) control; (**B**) 1 ng/mL FSH; (**C**) 5 ng/mL FSH; (**D**) 10 ng/mL FSH; (**E**) 25 ng/mL FSH; and (**F**) 50 ng/mL FSH. Granulosa Cells were challenged after 24 h of culture with the treatments given for 48 h in the left panel **(G)** or were challenged with 25 ng/mL FSH or not with FSH for the time given in right panel **(H)**. Cell viability assay was done by using CCk-8 assay kit after treatment and data are represented as mean ± S.E. of five independent cultures. Each panel without common letters is significantly different (*p* < 0.05).

Initially, the effect of FSH was observed on the viability of GCs from bovine follicles, acquired from a local abattoir. After 24 h of culture development, GCs were incubated at the rate of 1, 5, 10, 25, and 50 ng/mL FSH for 48 h along with control (no FSH) (Figure 3G). Significant high viability was found by the addition of 10 and 25 ng/mL FSH as compared to control (*p* < 0.05), while no significant difference was observed between 1 or 5 ng/mL FSH and control. Markedly, at 50 ng/mL FSH, significantly low viability was detected as compared to the untreated group.

To evaluate the time course of FSH treatment on cell viability, GCs were treated with 25 ng/mL FSH at different time points, i.e., 0, 6, 12, 24, and 48 h. GCs viability increased in a time-dependent manner, and it was highest at 24 h, while a decreasing trend was observed at 48 h (Figure 3H).

**Figure 4:**
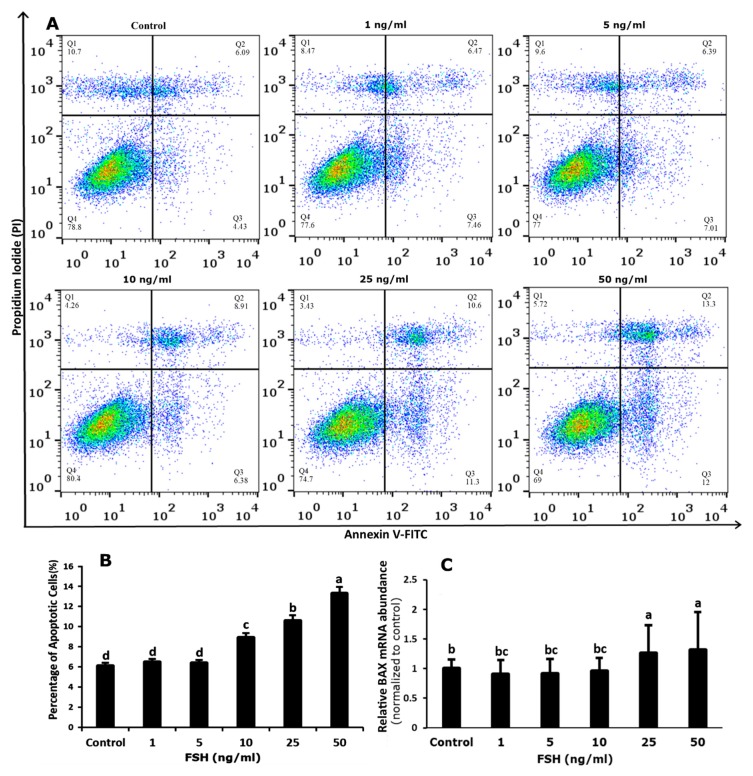
Effects of Follicle Stimulating Hormone (FSH) on the apoptosis in bovine granulosa cells. Granulosa Cells were challenged with increasing doses of FSH for 48 h starting on day two of culture. (**A**) The apoptotic cells were detected by flow cytometry. (**B**) Quantitative result of granulosa cells apoptosis as shown in A, ** *p* < 0.01. (**C**) Abundance of mRNA encoding *BAX*, * *p* < 0.05. Data are means ± SEM of five independent replicates. For each treatment, means without common letters are significantly different (*p* < 0.05). *BAX* = Bcl-2-associated X Protein.

We then evaluated the qualitative and quantitative effects of FSH on apoptosis in GCs. Quantitative study revealed that the highest significant number of apoptotic cells were found after addition of 50 ng/mL FSH as compared to control, then 25 and 10 ng/mL FSH respectively. However, none of the other doses (1 and 5 ng/mL FSH) altered the rate of apoptosis (Figure 4A,B). We also measured *BAX* mRNA abundance, which revealed that various FSH concentrations had different impacts on *BAX* mRNA abundance. For instance, *BAX* mRNA abundance was slightly decreased at lower doses of FSH (1 and 5 ng/mL) but increased significantly at 25 and 50 ng/mL, as compared to control (Figure 4C).

### 3.5. Quantification of Hormones in Follicular Fluid and Culture Medium

**Figure 5:**
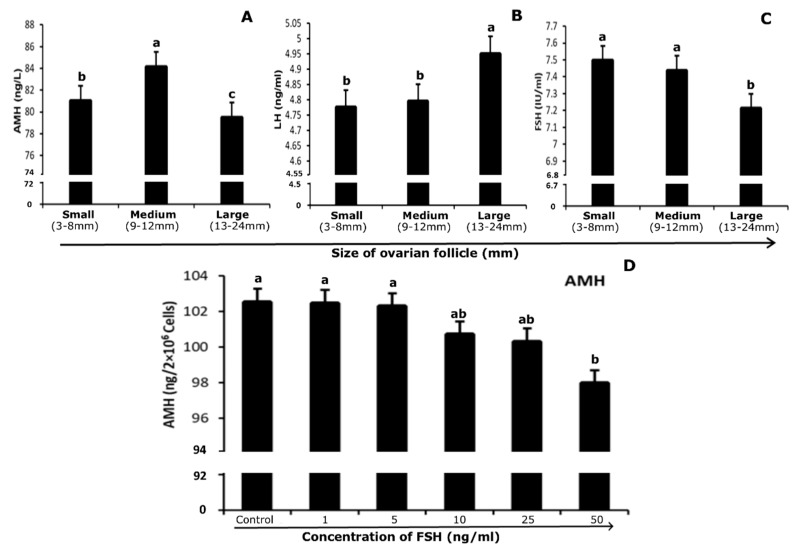
Concentration of AMH (ng/mL; **A**), FSH (mIU/ml; **B**) and LH (ng/mL; **C**) in follicular fluid of different size follicles. (**D**) AMH secretion (ng/2 × 10^6^ cells) in cultured media with increasing treatment of FSH (0, 1, 5, 10, 25, and 50 ng/mL). Data are represented as mean ± S.E. Means without common letters are significantly different (*p* < 0.05; *n* = 5 per follicle size or independent five culture medium). FSH = Follicle Stimulating Hormone. LH = Luteinizing Hormone. AMH = Anti-Mullerian Hormone.

Pooled follicular fluid was separated from GCs by centrifugation and used to quantify the level of AMH, FSH, and LH in a group of follicles of different sizes. The level of AMH in follicular fluid of different size follicles of 3–8 mm, 9–12 mm and 13–24 mm were 81.03 ± 5.81, 84.14 ± 5.41, and 79.49 ± 2.48 ng/mL, respectively. The concentration of AMH in follicular fluid was highest at 9–12 mm follicles than in 3–8 mm while the lowest level was found in 13–24 mm size follicles (*p* < 0.05; Figure 5A). In the follicular fluid, the concentration of FSH in different sized follicular groups, 3–8 mm, 9–12 mm and 13–24 mm, were 7.49 ± 0.26, 7.44 ± 0.26, and 7.21 ± 0.14 (mIU/ml), respectively. FSH revealed a gradually decreasing trend with increasing follicular diameter, 3–8 mm, 9–12 mm size follicles showed highest level of FSH protein and 13–24 mm follicles showed lowest concentration in follicular fluid (*p* < 0.05; Figure 5C). The amount of LH in follicular fluid at different sizes (i.e., 3–8 mm, 9–12 mm and 13–24 mm), large follicles (13–24 mm) showed the highest level of LH in contrast to small and medium ones (*p* < 0.05; Figure 5B). 

After culturing GCs (2 × 10^6^ cells per well), the medium was stored at −80 °C for the quantification of AMH. AMH level decreased gradually after the 5 ng/mL FSH dose. The minimum level was found at 50 ng/mL FSH treatment medium, while 1 and 5 ng/mL doses showed no significant difference as compared to control (*p* < 0.05; Figure 5D).

### 3.6. Association Between mRNA Abundance and Hormone (Protein) Level

**Figure 6:**
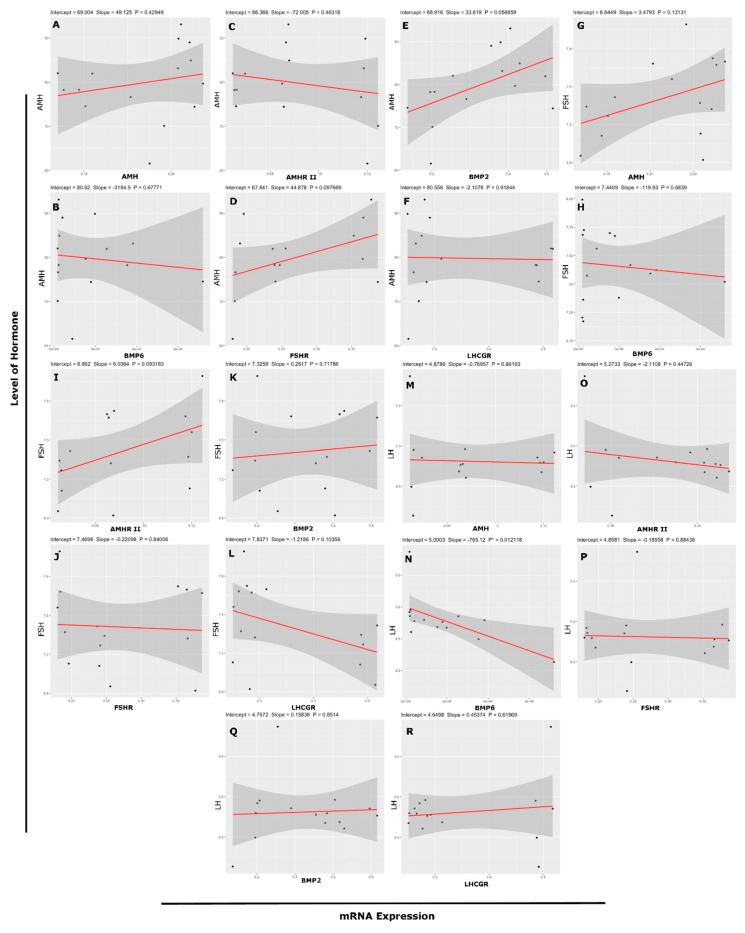
Correlation with linear regression model among level of hormone in follicular fluid and transcript abundance of genes. Relationship between (**A**) AMH level and mRNA expression of *AMH*, (**B**) AMH level and mRNA expression of *BMP6*, (**C**) AMH level and mRNA expression of *AMHR-II*, (**D**) AMH level and mRNA expression of *FSHR*, (**E**) AMH level and mRNA expression of *BMP2*, (**F**) AMH level and mRNA expression of *LHCGR*, (**G**) FSH level and mRNA expression of *AMH*, (**H**) FSH level and mRNA expression of *BMP6*, (**I**) FSH level and mRNA expression of *AMHR-II*, (**J**) FSH level and mRNA expression of *FSHR*, (**K**) FSH level and mRNA expression of *BMP2*, (**L**) FSH level and mRNA expression of *LHCGR*, (**M**) LH level and mRNA expression of *AMH*, (**N**) LH level and mRNA expression of *BMP6*, (**O**) LH level and mRNA expression of *AMHR-II*, (**P**) LH level and mRNA expression of *FSHR*, (**Q**) LH level and mRNA expression of *BMP2*, (**R**) LH level and mRNA expression of *LHCGR*, (* *p* < 0.05, ** *p* < 0.001). AMH = Anti-Mullerian Hormone. FSH = Follicle Stimulating Hormone. LH = Luteinizing Hormone. *FSHR* = Follicle Stimulating Hormone Receptor. *LHCGR* = Luteinizing Hormone/Choriogonadotropin Receptor. *AMHR-II* = Anti-Mullerian Hormone Receptor type-II. *BMP2* = Bone Morphogenetic Protein 2. *BMP6* = Bone Morphogenetic Protein 6.

The association between hormone level (AMH, FSH and LH) in follicular fluid and transcript abundance (*AMH, AMHR II, BMP2, BMP6, FSHR,* and *LHCGR*) in GCs shown in Table 2. The concentration of AMH in follicular fluid was positively associated with mRNA *FSHR* abundance (*r* = 0.44, *r*^2^ = 0.1967; Figure 6D). The transcript abundance of *AMH* (*r* = 0.41, *r*^2^ = 0.1663; Figure 6G) and *AMHR-II* (*r* = 0.45, *r*^2^ = 0.2015; Figure 6I) was positively associated with levels of FSH, and there was a lower linear relationship between them. Meanwhile, transcript *LHCGR* abundance showed a negative association with the concentration of FSH (*r* = −0.44, *r*^2^ = 0.1970; Figure 6L). The transcript *BMP6* abundance and LH level showed a significant and negative relationship, but 39.48% data showed the linear relationship between both variables (*r* = −0.63, *r*^2^ = 0.3948; Figure 6N). None of the other variables showed any relationship between them. Figure 6 illustrates the relationship according to regression analysis between the level of hormone in follicular fluid and transcript abundance of genes. 

**Figure 7:**
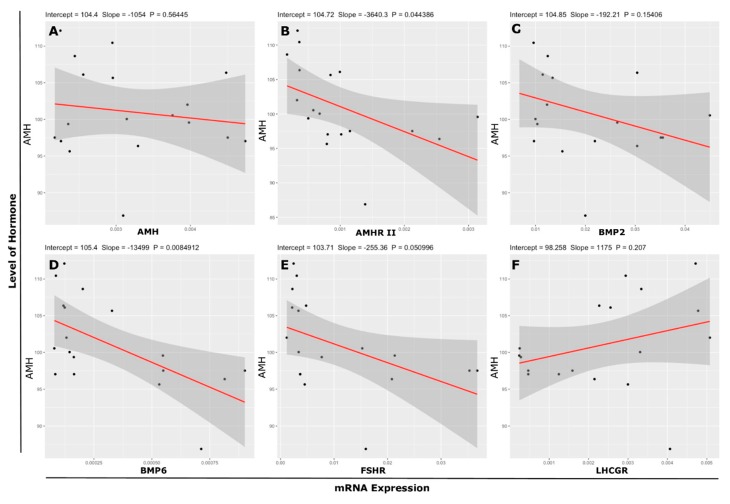
Relationship according to regression line between level of hormone in culture medium and transcript abundance of genes. Relationship between (**A**) AMH level and mRNA expression of *AMH*, (**B**) AMH level and mRNA expression of *AMHR-II*, (**C**) AMH level and mRNA expression of *BMP2*, (**D**) AMH level and mRNA expression of *BMP6*, (**E**) AMH level and mRNA expression of *FSHR*, (**F**) AMH level and mRNA expression of *LHCGR*, (* *p* < 0.05, ** *p* < 0.01, *** *p* < 0.001). AMH = Anti-Mullerian Hormone. *FSHR* = Follicle Stimulating Hormone Receptor. *LHCGR* = Luteinizing Hormone/Choriogonadotropin Receptor. *AMHR-II* = Anti-Mullerian Hormone Receptor type-II. *BMP2* = Bone Morphogenetic Protein 2. *BMP6* = Bone Morphogenetic Protein 6.

An association among the transcript abundance (*AMH, AMHR-II, BMP2, BMP6, FSHR* and *LHCGR*) of cultured undifferentiated GCs collected from 3–8 mm follicles and the level of AMH in culture media shown in Table 3. The level of AMH in culture medium exhibited a negative but significant association with mRNA abundance of *AMHR-II* (*r* = −0.48, *r*^2^ = 0.2293; Figure 7B) and a 22.93% variation in AMH can be explained the linear relationship between the AMH and *AMHR-II*. Furthermore, a 22.93% regression line represents the data. *BMP2* (*r* = −0.35, *r*^2^ = 0.1227; Figure 7C) showed a negative association with AMH and a 12.27% linear relationship. *BMP6* (*r* = −0.60, *r*^2^ = 0.3599; Figure 7D) abundance showed a highly significant negative association with AMH and 35.99% variance was calculated between both variables. Furthermore, positive association was found between level of AMH in culture medium and mRNA *LHCGR* abundance (*r* = 0.31, *r*^2^ = 0.0975; Figure 7F) but variance is very low (9.75%) between variables. A linear relationship according to regression analysis among the concentration of AMH in media and mRNA abundance of *AMH, AMHR II, BMP2, BMP6, FSHR*, and *LHCGR* is described in Figure 7A–F, respectively.

## 4. Discussion

The molecular mechanisms regulating follicular growth and dominance in mono-ovulatory species have not been completely elucidated. However, AMH is shown to be expressed in GCs [11], and the regulation of AMH and its receptor *AMHR-II* has not been systematically investigated during follicular deviation and the selection of the dominant follicle in mono-ovulatory species. In this study, cow ovaries were used to investigate the regulation of AMH and its receptor *AMHR-II* at follicular, cellular, and endocrine levels and in response to FSH supplementation in vitro. The mRNA *AMH* level was found to be higher in bovine GCs onset to follicle deviation and significantly decreased after selection of dominant follicle by using follicular selection and deviation model during folliculogenesis [33,34,35]. Other studies observed a similar trend in the follicular fluid concentration of AMH during follicle deviation and selection. Our findings are in accordance with the previous research in bovines, reflecting the highest level of AMH from pre-antral to early antral stage follicles [11,36,37,38]. In rats, the expression of *AMH* has been described to be decreased in the final stage of follicle development [39]. Our findings are in agreement with already reported studies in women, revealing a decline trend of *AMH* expression in GCs after follicle selection and deviation [40,41,42]. On behalf of the aforementioned studies, we propose that mRNA abundance and the concentration of AMH in GCs are similarly regulated during follicle selection and deviation in mono and poly-ovulatory species. In the present study, the transcript abundance of *AMH* and *AMHR-II* were in same manner. *AMHR-II* also has high expression before the selection of the dominant follicle, while the highest level of expression was in pre and early antral follicle GCs. These findings are in line with the finding from cattle ovaries, collected from the slaughterhouse, presenting lower levels of *AMH* mRNA in atretic follicles as compared to healthy follicles [10,36]. All these studies mentioned above propose that *AMH* abundance might be regulated by FSH during follicle growth. Our study was also conducted at the in vitro GC level with undifferentiated GCs isolated from 3–8 mm follicle size and culture under the different supplementary doses of FSH (1, 5, 10, 25 and 50 ng/ul) along with control. The mRNA expression of its cognate FSH receptor also increased which confirms the success of treatment under high FSH concentrations. *AMH* mRNA abundance was most significantly increased by 25 ng/mL FSH and 10 ng/mL also showed a slight increase in mRNA abundance, while extreme lower or higher concentrations had a non-significant impact on the mRNA abundance of *AMH*. Similarly, *AMHR-II* mRNA abundance was most significantly enhanced at 10 ng/mL FSH, followed by 5 and 25 ng/mL FSH, while other FSH concentrations had a less significant impact on the mRNA abundance of *AMHR-II*. Our results coincide to the findings of in vitro GC cultures [43,44] and are also in contrast to the in vivo findings of Ilha et al. [37]. FSH has an inhibitory effect on *AMH* and *AMHR-II* transcript abundance in rats at the time of follicle deviation from early antral to late/large antral follicles [39]. Additionally, FSH signaling decreases the abundance of *AMH* in chicken GCs [45] and serum concentration in humans [46]. The versatile role of FSH on AMH production between all these reports may be ascribed to the stage of follicle, source of GCs, and/or length or duration of culture [38,47]. Furthermore, Durlinger et al. found AMH suppress the *FSHR* and aromatase expression in rat and murine GCs respectively [47,48,49]. Similarly, Chang et al. [50] found that AMH declines the *CYP19A1* expression and ultimately estrogen synthesis in human granulosa-lutein cells.

Additionally, a stimulatory role of BMPs on *AMH* abundance has been established in human, poultry, bovine and ovine GCs [22,38,45,51,52]. Due to the significance of BMP system in regulating the reproductive system, further studies have emphasized the molecular mechanism, revealing the involvement of BMPs in the ovarian process [53]. During folliculogenesis, the abundance of *BMP2* increases in GCs of early antral follicles. Given the results of our research are according to the findings of Diaz et al. [54], the expression of *BMP2* in GCs was higher in antral follicles [54]. The expression pattern is concordant with the previously illustrated expression pattern of *BMP2* in the ovarian somatic cells [24]. In vitro, *BMP2* expression increased under the treatment of FSH, as reported by Glister et al. [55]. In vitro, *BMP6* expression did not exhibit a specific manner, similarly to *AMH, AMHR-II,* and *BMP2* expressions observed in in vitro culturing of GCs from 3–8 mm follicles. Unlike BMP2, BMP6 does not support FSH stimulation [56,57]. Our results also depict a varying expression trend of *BMP2* and *BMP6* in vitro. Other studies also show that BMP6 has an inhibitory role during in-vivo folliculogenesis, i.e., AMH inhibits GC proliferation and later ultimately reduces aromatase expression [38,48]. FSH decreases the cell cycle and suppresses mitophagy via the PINK1-Parkin (PTEN-induced kinase 1) cascade to promote GC survival [58,59]. Initially, Cell viability increased with the concentration of FSH, but significantly decreased at high doses (50 ng/mL FSH). Multiple biological functions of FSH have been demonstrated. Nuclear factor-kappa B (NF-κB) is a nuclear transcription factor that regulates the expression of a large number of genes that are critical for the regulation of apoptosis [60]. After PKa (protein kinase a) activates FSH, gsK-3β (glycogen synthase kinase 3 beta) activity is downregulated, which induces the inactivation of the NF-κB pathway to activate cell apoptosis [58]. As per our findings, apoptosis markedly increased at 25 and 50 ng/mL FSH doses as compared to control. 

The level of AMH in intrafollicular fluid in bovines are reported variable ranged from 400 to 800 ng/mL in 3–5 mm follicles, but to a lesser extent in in 7–10 mm follicles (approximately 200–400 ng/mL) [10,36,37,48,61]. In this study, our AMH concentration is near to the above-mentioned level of AMH. In-vitro, AMH protein levels showed a significant negative association with mRNA *AMHR-II*. However, a similar relationship was also reported by Ilha et al. [37]. In contrast, many studies in humans showed positive associations between mRNA *AMH* of GCs and hormone levels of AMH in follicular fluid [42]. In bovines, the concentration of AMH was decreased with an increasing size of follicle, while a difference in transcript level of GC is not always parallel with the hormone concentration of follicular fluid [10,36]. AMH inhibits FSH-dependent follicle growth and estrogen production in follicles, while BMP6 has the opposite effect [56,57]. As per the above discussion, BMPs play an important role at the FSH–AMH nexus in bovine GCs to modulate folliculogenesis [48,55].

## 5. Conclusions

In the present study, our findings revealed that *AMH*, *AMHR-II* and *BMP2* mRNA levels increased before follicle deviation and selection, whereas they decreased after the follicle deviation. During short term exposure (48 h), low doses of FSH increased the transcript abundance of *AMH*, *AMHR-II*, and *BMP2*. We found an optimum level of FSH (25 ng/mL) which can significantly enhance *AMH* and *BMP2* abundance. The relationship between mRNA expression and protein level appeared to be quite variable among in vivo and in vitro studies, which might be due to the involvement of autocrine and paracrine factors. Further in vivo field studies using large animal groups are necessary to elucidate this antagonistic relationship between FSH and AMH, and its implications towards the improvement of fertility and ART outcomes in bovines.

## Figures and Tables

**Table 1:** Primers sequence for qRT-PCR.

Gene Symbol	Gene ID.	Oligo	Primer Sequence (5′–3′)
AMH	NM_173890	Forward Reverse	CAGGGAAGAAGTCTTCAGCA AAGGTGGTCAAGTCACTCAG
AMHR ll	NM_001205328	Forward Reverse	AGCTGTGCTTCTCCCAGGTCATA AAAGCGGACAATGTGGTCATGCTG
BMP2	NM_001099141	Forward Reverse	TGTCCAAACTCTGGTCAACTC CTCGACAACCATGTCCTGATAG
BMP6	XM_005192966	Forward Reverse	CGCCAGCGACACCACAAAGAGT CCCAGCAGAAACAGGTCCGAGTC
CYP19A1	NM_174305.1	Forward Reverse	GGCTATGTGGACGTGTTGACC TGAGAAGGAGAGCTTGCCATG
FSHR	NM_174061	Forward Reverse	AGGCAAACGTGTTCTCCAAC CGGAGGTTGGGAAGGTTCTG
LHCGR	NM_174381	Forward Reverse	TGAACTGAGTGGCTGGGATT AGGACAGTCACATTTCCCGT
BAX	NM_173894	Forward Reverse	AACATGGAGCTGCAGAGGAT CAGTTGAAGTTGCCGTCAGA
GAPDH	NM_001034034.1	Forward Reverse	CACCCTCAAGATTGTCAGCA GGTCATAAGTCCCTCCACGA
ACTB	NM_173979.3	Forward Reverse	GTGACATCAAGGAGAAGCTCTG TTGAAGGTAGTTTCGTGAATGC

**Table 2:** Correlation between hormone levels and mRNA expression in different size follicles.

mRNA Expression ›		AMH	AMHR II	BMP2	FSHR	LHCGR	BMP6
**AMH (ng/mL)**	r value	0.22	−0.21	0.5	0.44	−0.03	−0.12
	*p* value	0.4295	0.4632	0.0569	0.0977	0.9184	0.6777
	r^2^ value	0.0486	0.0421	0.2514	0.1967	0.0008	0.0137
	AdjR^2^ value	−0.0245	−0.0316	0.1938	0.1349	−0.0760	−0.0621
**LH** (ng/mL)	r value	−0.21	−0.05	0.05	−0.04	0.14	−0.63 *
	*p* value	0.4473	0.8619	0.8514	0.8844	0.6197	0.0121
	r^2^ value	0.0451	0.0024	0.0028	0.00168	0.0194	0.3948
	AdjR^2^ value	−0.0283	−0.0743	−0.0739	−0.0751	−0.0559	0.3482
**FSH** (mIU/mL)	r value	0.41	0.45	0.1	−0.06	−0.44	−0.11
	*p* value	0.1313	0.0932	0.7179	0.8401	0.1036	0.6839
	r^2^ value	0.1663	0.2015	0.0104	0.0032	0.1907	0.0132
	AdjR^2^ value	0.1022	0.1401	−0.0657	−0.0734	0.1285	−0.0627

Significant * *p* < 0.05, highly significant ** *p* < 0.001.

**Table 3:** Correlation relationship with regression analysis among the expression of culture GC genes and AMH levels in media.

mRNA Expression ›		AMH	AMHR-II	FSHR	LHCGR	BMP2	BMP6
AMH (ng/2 × 10^6^ cells)	r value	−0.15	−0.48 *	−0.47	0.31	−0.35	−0.60 **
	*p* value	0.5645	0.0444	0.051	0.207	0.1541	0.0085
	r^2^ value	0.0212	0.2293	0.2176	0.0975	0.1227	0.3599
	AdjR^2^value	−0.0399	0.1811	0.1687	0.0411	0.0679	0.3198

Significant * *p* < 0.05, highly significant ** *p* < 0.001.
